# The Impact of Technostress Generated by Artificial Intelligence on the Quality of Life: The Mediating Role of Positive and Negative Affect

**DOI:** 10.3390/bs15040552

**Published:** 2025-04-19

**Authors:** Daniela-Elena Lițan

**Affiliations:** Psychology Department, West University of Timișoara, 300223 Timișoara, Romania; daniela.litan@e-uvt.ro

**Keywords:** artificial intelligence, technostress, quality of life, positive affect, negative affect

## Abstract

In the era of Artificial Intelligence, the magic of achieving results at the “speed of light” for tasks that until recently required a lot of work and effort shocks, arouses enthusiasm and generates fears at the same time. Therefore, starting from this reality of our days, we proposed within the current research to study the relationship between the factors of technostress (techno-overload, techno-invasion, techno-complexity, techno-insecurity, techno-uncertainty) perceived as a result of the implementation of AI at the societal level and the quality of life, filtering the relationship through the “lens” of the positive and negative affect mediators. The mediation analyses, conducted on a sample of 217 adult Romanian citizens (18–62 years old), suggested that although AI-related technostress does not directly influence quality of life, it has a significant indirect impact through affective traits—general tendencies to frequently experience positive or negative emotions. This indicates that technostress contributes to variations in quality of life by influencing emotional experiences, which mediate the relationship. These findings emphasize not only the absence of a direct effect, but also the importance of the indirect pathway in understanding how individuals are affected by AI-related stress. We believe that the results of the current study can be equally useful in raising awareness of the psychological mechanisms responsible for the quality of life and in understanding the importance of implementing official programs, both technically, regarding the development of skills to understand and work with AI, and psychological support programs, considering the management of emotions, with reference to this technology.

## 1. Introduction

Artificial Intelligence (AI) systems are becoming an increasingly important part of our lives, as they have the capacity to demonstrate human-like abilities, such as reasoning, learning, planning, and creativity (European Parliament, 2023). Although AI promises efficiency and innovation through scalability, reliability, precision, speed, decision-making, real-time monitoring, robotic assistance in performing tasks, and optimal use of resources (Sharma, 2024), its accelerated pace of development also generates significant challenges. AI also has disadvantages, including high costs for creating AI machines, lack of creativity, replacement of jobs with robots, diminishing human involvement in various activities, especially professional ones, and many others (Bhbosale et al., 2020; Stevie & Ammara, 2023). However, perhaps the greatest challenge felt today, when AI technology is still in development, without having reached its true potential, is related to the human psyche, which is ready to project often frightening scenarios, generating stress and anxiety, but also hostility regarding the implementation of AI at a societal level. An increasingly common and studied phenomenon is technostress in relation to AI technology and the continuous adaptation to these technological innovations. Studies suggest that technostress can have a negative impact on mental health (Borle et al., 2021; Consiglio et al., 2023; Sommovigo et al., 2023) and on the quality of life (Bottaro et al., 2025; Mehmood et al., 2024).

Technostress is defined as the stress experienced by end users in organizations as a result of using IT products, namely, as an important disadvantage of information technology (Bondanini et al., 2020). Nevertheless, technostress can also involve positive challenges that enhance performance and learning—a phenomenon referred to as techno-eustress (Califf et al., 2020; Nascimento et al., 2024). The present study, however, focuses specifically on the negative and detrimental aspects of technostress (also known as techno-distress). The harmful impact of technostress on individuals includes several psychological and emotional consequences, as highlighted by Porcari et al. (2023), such as burnout (Consiglio et al., 2023; Wirth et al., 2024), depression and anxiety (Galvin et al., 2022; Torales et al., 2022), as well as a persistent pressure to remain constantly available or connected, which affects areas such as work–family balance (Carlotto et al., 2017) and the ability to manage multiple tasks simultaneously (Saleem & Malik, 2023).

Recent studies have also employed mediation models to examine how technostress influences psychological outcomes through intermediate mechanisms. For example, several works have identified the mediating role of work–family conflict or balance (Gemmano et al., 2023), as well as mood regulation or emotional states (Ali et al., 2023; Jung, 2013), in the relationship between technostress and burnout. These findings underscore the importance of investigating affective variables as potential mediators—a direction also adopted in the present study.

Also, following the installed technostress, the specialized literature reports other consequences, such as cognitive overload and reduced performance (EL Kiassi & Jahidi, 2023; S. Y. Kim et al., 2022).

Technostress (Technostress creators) consists of five main factors (Ragu-Nathan et al., 2008): techno-overload, techno-invasion, techno-complexity, techno-insecurity and techno-uncertainty. These factors generate stress and burnout (Tarafdar et al., 2007; Wirth et al., 2024), thus affecting the emotional health of individuals.

In the specialized literature, we find technostress associated with technology represented by smartphones (Boonjing & Chanvarasuth, 2017; Hämäläinen et al., 2024; Yao & Wang, 2023), social networks (Brooks & Califf, 2017; Naga & Ebardo, 2025; Saini & Phoolka, 2024) and generally associated with the field of ICT (Information and Communication Technology) (Salazar-Concha et al., 2021; Saleem et al., 2024; K. Wang et al., 2008). The association between technostress and AI (Chang et al., 2024; Issa et al., 2024; Zhang & Tong, 2024) is found especially in the literature of recent years, but studies are still insufficient to fully understand the effects of AI on mental health.

Quality of life, in turn, reflects the physical, mental and social well-being of the individual, as well as their ability to carry out their daily activities in a harmonious and balanced manner (Paşcanu et al., 2009). The reduction of positive affect and the increase of negative affect significantly influence important areas of life, including mental health, professional performance and social relationships (Eryilmaz et al., 2023; Plys & Desrichard, 2020; Versteeg et al., 2009; K. Wang et al., 2024).

However, little is known about how technostress specifically triggered by AI affects individuals’ quality of life, particularly through emotional mechanisms. To address this gap, the present study explores both the emotional and psychological processes involved in this relationship. The current research examines the mediating role of positive and negative effects in the relationship between technostress triggered by AI technology and the quality of life, starting from several psychological theories. To support this approach, the study is grounded in three key psychological frameworks that offer a comprehensive understanding of how technostress may influence quality of life through affective mechanisms. The first framework is the transactional theory of stress and coping (Lazarus & Folkman, 1984), which posits that a person’s stress reactions and coping strategies depend on how they evaluate the situations they face. In this perspective, coping is defined as a dynamic process of cognitive and behavioral adjustment, through which the individual tries to manage external or internal demands perceived as imposing or exceeding personal resources (Spaderna & Hellwig, 2015). Therefore, we believe that this theory can explain how individuals perceive and manage stress caused by technology.

The second framework is the Job Demand Control Model (Karasek, 1979). The central concept of this model is that an adequate level of employee control can reduce the impact of job demands on effort and can contribute to increasing employee satisfaction by providing them with the opportunity to take on challenging tasks and develop new skills (Kain & Jex, 2010). Therefore, high levels of technical demands combined with low environmental control can increase the employees’ stress.

The third theoretical framework is the Positive and Negative Affect Theory (Watson & Tellegen, 1985). According to this theory, a high level of positive affect is manifested by enthusiasm, energy and alertness, reflecting an active and optimistic mood. In contrast, negative affect is associated with aversive emotions, such as anger, guilt, nervousness and fear, highlighting states of tension and emotional discomfort (Fortenberry et al., 2017). Therefore, we believe that based on this theory, it is possible to explain how emotional reactions can mediate the effects of stress on the quality of life. In this context, technostress can increase the levels of negative affect (anxiety, frustration, exhaustion) and reduce positive affect (satisfaction, enthusiasm).

Given the context presented above, it is reasonable to consider that constant exposure to technology, without adequate coping mechanisms, may have a significant negative impact on the overall well-being of the individual. Therefore, the present research focuses on the adult population with Romanian citizenship and aims to examine the mediating role of positive and negative affect in the relationship between technostress generated by AI technology and the quality of life. In line with this objective, a set of hypotheses has been developed to explore these mediation effects in depth, as follows:

**H1.**  *Positive affect mediates the relationship between AI-generated technostress and quality of life. This is a negative mediation, meaning that higher levels of technostress lead to lower levels of positive affect, which in turn reduces quality of life. Each technostress factor is tested separately: H1.1: Techno-overload, H1.2: Techno-invasion, H1.3: Techno-complexity, H1.4: Techno-insecurity, H1.5: Techno-uncertainty.*

**H2.**  *Negative affect mediates the relationship between AI-generated technostress and quality of life. This is also a negative mediation, as higher levels of technostress lead to higher levels of negative affect, which in turn reduces quality of life. Each technostress factor is tested separately: H2.1: Techno-overload, H2.2: Techno-invasion, H2.3: Techno-complexity, H2.4: Techno-insecurity, H2.5: Techno-uncertainty.*

**Figure 1:**
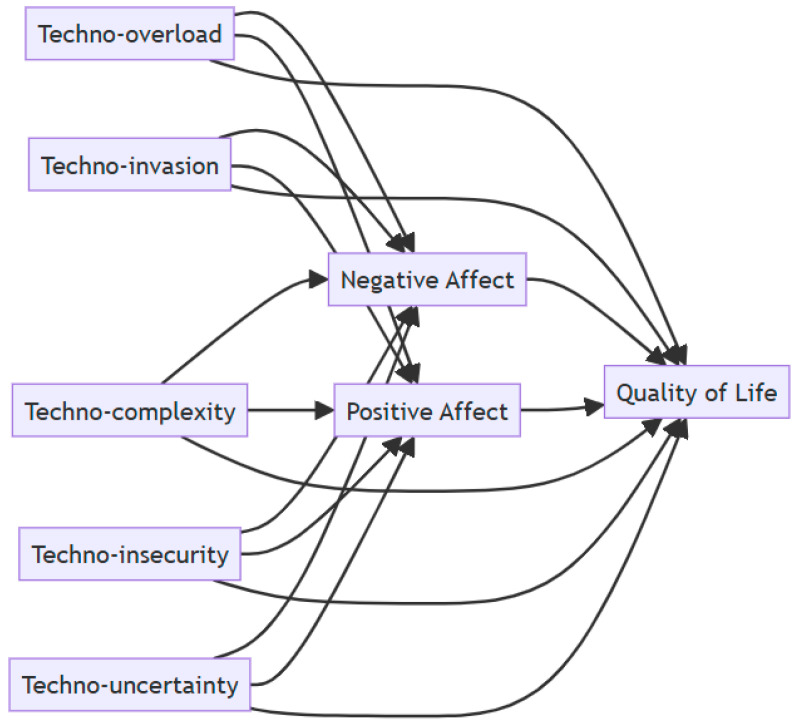
The hypothesized model.

In Figure 1, we can see the previously proposed theoretical model.

## 2. Methods

### 2.1. Study Design

The current study, conducted in accordance with the World Medical Association Helsinki declaration, was approved by the Scientific Council of the University Research and Creation from the West University of Timișoara, Romania, in August 2024 (process number: 53168).

The current research has a cross-sectional survey design and the responses to the questionnaires used were collected online, using the Google Forms platform, between 11 December 2024 and 8 February 2025.

The participants in the study were also informed about its context, objective, and purpose, and were all provided with informed consent.

### 2.2. Participants

The study was conducted on 217 individuals (37.8% men and 62.2% women), Romanian citizens, adults, aged between 18 and 62 years (M = 36,15, SD = 11.92), whose level of education (last school graduated) ranged from high school—24%, college—35.9%, master’s degree—35% to doctorate/post-doctorate—5.1%. The professional status also ranged from employees—65.9%, students—18.4%, self-employed—12.4% to unemployed (1.4%), retired (0.9%) and housewives (0.9%).

The participants in the study live in both urban (80.1%) and rural (19.9%) areas; 70.5% are in a couple relationship and 55.1% of them do not have children.

The selection criteria for participants for inclusion in the study were Romanian citizenship, age between 18 and 65 years, and the ability to understand a written text. All respondents self-identified as either male or female.

The conditions for excluding the responses received from participants were the participant’s refusal to give consent to participate in the study and abandoning the questionnaire; that is, not completing it in full.

The batch size was calculated a priori with the G*Power program, which for an average effect, a power of 0.80, a type I error equal to 0.05 and 5 predictors displayed a minimum size of 92 participants.

### 2.3. Measures

*(a)*  The Technostress creators scale

The technostress is a multidimensional phenomenon closely related to the use of information and communication technologies (Porcari et al., 2023). More precisely, technostress is a problem of adaptation experienced by individuals when they are unable to cope with the challenges associated with the use of technology (X. Wang et al., 2020). In the case of the current study, technology is represented by AI. The technostress scale consists of two components (Ragu-Nathan et al., 2008): Technostress creators (measures stress-generating factors in relation to technology) and Technostress inhibitors (measures factors that have the potential to reduce the effects of technostress). In the current study, the Technostress creators component was used in relation to the use of AI. Other studies with Romanian participants have also used this scale, proving very good reliability (Cazan et al., 2024; Truța et al., 2023).

The Technostress creators scale consists of 23 items, which describe the 5 factors of technostress (Ragu-Nathan et al., 2008):

- Techno-overload (6 items) refers to situations where the use of AI technology causes users to increase their work pace and workload.
- Techno-invasion (3 items) refers to situations where users remain constantly connected to technology (AI), unable to separate their personal and professional spheres.
- Techno-complexity (5 items) refers to situations where users must invest significant time and effort to learn and understand how technology (AI) works.
- Techno-insecurity (5 items) refers to situations where people perceive technology (AI) as a threat to their jobs, either because of the automation of human activities or because of competition with people who are more skilled in using AI.
- Techno-uncertainty (4 items) represents situations in which the rapid and continuous evolution of technology (AI) generates instability and uncertainty, forcing people to constantly adjust and learn.

Although the technostress model developed by Ragu-Nathan et al. (2008) is not recent, it remains one of the most comprehensive and widely adopted frameworks in the field. The five dimensions it proposes—techno-overload, techno-invasion, techno-complexity, techno-insecurity, and techno-uncertainty—offer a nuanced structure that is highly relevant for analyzing the stress generated by rapidly advancing technologies, including AI. Given the conceptual clarity and consistent use of this model in more recent studies on digital technologies (e.g., Bottaro et al., 2025; Califf et al., 2020; Tarafdar et al., 2019), it was considered the most appropriate choice for the objectives of the current research.

The Technostress creators questionnaire allows participants to record responses on a 5-point Likert scale (from 1-Totally Disagree, to 5-Totally Agree).

The five factors of the Technostress creators questionnaire used in the current research had good internal consistency with Cronbach’s alpha values: 0.881 (Techno-overload), 0.810 (Techno-invasion), 0.847 (Techno-complexity), 0.830 (Techno-insecurity), 0.907 (Techno-uncertainty).

*(b)*  Positive and Negative Affect Scales, extended form—PANAS-X

The PANAS-X scales are a 60-item self-report instrument that describes different feelings and emotions experienced in the past few weeks (Watson & Clark, 1994). The instrument measures both General Positive Affect and General Negative Affect, as well as 11 specific affects: fear, sadness, guilt, hostility, shyness, fatigue, surprise, joviality, self-confidence, attention, and calmness (Cotigă, 2012). In the current study, only the factors General Positive and Negative Affect have been used, each consisting of 10 items.

The PANAS-X scales allow participants to record responses on a 5-option Likert scale (from 1- very slightly or not at all, to 5- extremely).

The two factors of the PANAS-X scale used in the current research had good internal consistency, with Cronbach’s alpha values of 0.891 (Negative Affect) and 0.843 (Positive Affect). The items were retained in the current research, according to the validated version of the scale, in Romanian (Cotigă, 2012).

*(c)*  Quality of Life Scale—QOLS

The Quality of Life Scale was originally created by the American psychologist John Flanagan in the 1970s, and was later adapted in a revised version (Burckhardt & Anderson, 2003). In their adaptation of the QOLS, Burckhardt and Anderson (2003) replaced the original 5-point response scales with a 7-point Likert-type scale ranging from “terrible” to “delighted.” This version was validated for use in American populations with chronic illness, aiming to improve sensitivity and psychometric performance. The revised version aimed to enhance response discrimination and better reflect the emotional range of respondents in a clinical context. The QOLS scale consists of five subscales: Material and Physical Well-Being, Relationships with Other People, Social, Community and Civic Activities, Personal Development and Fulfillment, and Recreation (Răscol, 2017). In the current study, the general value of the quality of life was used.

The QOLS scale consists of 16 items that allow participants to record responses on a 7-point Likert scale (from 1 = terrible, to 7 = delighted).

As far as the current research is concerned, the QOLS instrument had good internal consistency, with a Cronbach’s alpha value of 0.915.

It is also necessary to mention that the QOLS instrument has been successfully used in numerous doctoral theses and research studies conducted in Romanian, which confirms its suitability for this context; for example, (Albadi, 2021; Butuc, 2019; Frîncu, 2017; Hluhaniuc, 2017; Popovici et al., 2020; Răscol, 2017; Spinu et al., 2021).

### 2.4. Procedure

The study was pre-registered on the Open Science Framework platform (objectives, main hypotheses, study design, data collection procedure, measured variables and statistical analysis plan), before data collection. The pre-registration can be consulted at https://osf.io/uhkrw/?view_only=007c92b8febb438e9c664f2b2a25d80a, accessed on 5 December 2024 (the current study is a sub-study of the research on the theme “The intensity of the digital age” and its impact on the quality of life—emotional and psychosocial perspective of the individual).

The questionnaire link was shared on professional, social, and mobile messaging platforms and sent by email to the target groups. Participation was voluntary, anonymous, and without compensation.

The survey included demographic items (e.g., year of birth, gender, level of education, professional status, marital status) followed by the Technostress questionnaire (Technostress Creators Questionnaire; Ragu-Nathan et al., 2008), the Positive and Negative Affect Scales—extended form—PANAS-X (Watson & Clark, 1994) and the Quality of Life Scale—QOLS (Burckhardt & Anderson, 2003).

### 2.5. Statistical Analysis

Within the current research, the statistical analysis was performed using the Jamovi software, version 2.3.28.0 (The Jamovi Project, 2022). The modules used were the following:

- Descriptives, for displaying descriptive statistics;
- Reliability Analysis, for calculating the internal consistencies of the scales used in the research (Cronbach’s alpha values);
- Correlation Matrix, for analyzing correlations between variables;
- Principal Component Analysis, for common method bias analysis;
- Linear Regression—Collinearity statistics, for evaluating multicollinearity as part of the common method bias assessment;
- Path Analysis (via the pathj module), for computing R^2^ values of endogenous variables in the mediation model and evaluating the explanatory power of the full mediation structure, including both direct and indirect effects (Gallucci, 2021; Rosseel, 2012);
- Medmod—GLM Mediation Model, for moderated mediation and bootstrap 5000 analyses (Gallucci, 2020).

For the graphical display of the mediation model, the analysis was performed in RStudio, version 2024.12.0 (build 467), using the DiagrammeR package (Iannone & Roy, 2025), with Mermaid graph syntax, in R version 4.3.1 (R Core Team, 2023).

## 3. Results

### 3.1. Common Method Bias Analysis

The common method bias analysis was performed using the principal component analysis method from Jamovi. The results show that the first component (the Technostress creators questionnaire) explains 14.47% of the total variance, a value below the 50% threshold. According to previous recommendations, this result indicates that there is no significant risk of common method bias (Podsakoff et al., 2003).

The correlation matrix also shows that the highest inter-construct correlation is below 0.673 and no correlation exceeded the threshold of 0.90 (Bagozzi et al., 1991), indicating that there is no serious problem of common method bias. Furthermore, in order to detect multicollinearity, the analysis shows that the variance inflation factor (VIF) of all constructs is lower than the threshold of 5 (Hair et al., 2011; Kock, 2017), with the highest having the value of 2.145—Techno-overload. Also, tolerance is higher than 0.1 for all constructs. Therefore, multicollinearity is excluded as a significant source of common method bias.

### 3.2. Preliminary Analysis

The descriptive statistics and the correlations between the study variables can be seen in Table 1. Analyzing Table 1, we can notice that the quality of life variable is correlated with technostress factors, as follows: techno-invasion (r = −0.186, *p* < 0.01), techno-complexity (r = −0.205, *p* < 0.01), techno-insecurity (r = −0.216, *p* < 0.01). We also find, in Table 1, weak to moderate correlations between technostress factors and negative affect (techno-overload: (r = 0.288, *p* < 0.001), techno-invasion: (r = 0.347, *p* < 0.001), techno-complexity: (r = 0.317, *p* < 0.001), techno-insecurity: (r = 0.398, *p* < 0.001)) and positive affect (techno-complexity: (r = −0.135, *p* < 0.05)), but also strong correlations between positive affect (r = 0.638, *p* < 0.001), negative affect (r = −0.584, *p* < 0.001) and the quality of life. All these correlations identified in Table 1 are consistent with the previously formulated hypotheses, H1 and H2, and suggest possible associations between technostress, affect, and quality of life.

However, it is worth noting that not all correlations between technostress factors and quality of life are significant. Specifically, techno-overload and techno-uncertainty do not show statistically significant associations with quality of life. These non-significant correlations indicate that the support for the hypotheses H1 and H2 is only partial at this stage and that further mediation analyses are necessary to clarify these relationships.

### 3.3. The Relationship Between AI-Generated Technostress Factors and the Quality of Life, Mediated by Positive Affect and Negative Affect

In order to simplify the interpretation of the results, standardized z-scores of the predictors and the dependent variable were used.

All main analyses were performed through mediation modeling, as this allowed for a more theoretically grounded and nuanced exploration of the indirect effects of technostress on quality of life. The explanatory power of the overall mediation model was evaluated using a path analysis. The R^2^ values obtained for the endogenous variables were 20.6% for negative affect, 5.9% for positive affect, and 51.8% for quality of life. These values indicate a good model fit, especially in the case of quality of life.

**Figure 2:**
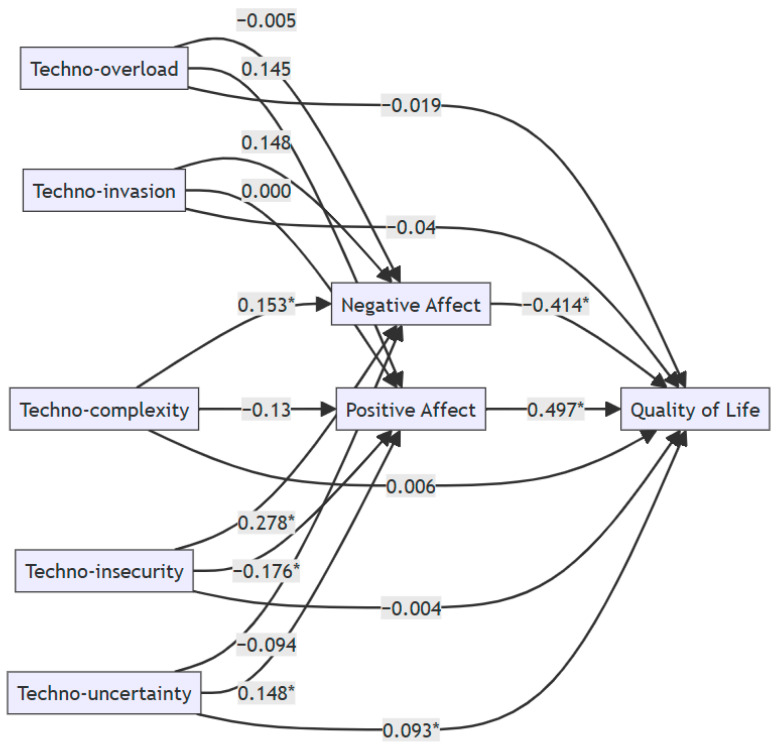
Estimated mediation model with standardized coefficients (β). Note. Asterisked (*) paths indicate statistically significant relationships.

Table 2 and Figure 2 present the results of the parallel mediation analysis examining the role of positive and negative affect in the relationship between technostress factors (techno-overload, techno-invasion, techno-complexity, techno-insecurity, techno-uncertainty) and quality of life.

(a) Positive affect as mediator:
  - Techno-uncertainty → quality of life: indirect effect β = 0.073, *p* < 0.05, 95% CI [0.004, 0.143]; complete mediation.
  - Techno-insecurity → quality of life: marginally significant indirect effect β = −0.087, *p* = 0.053, 95% CI [−0.176, 0.001]; mediation trend observed.
(b) Negative affect as mediator:
  - Techno-complexity → quality of life: indirect effect β = −0.063, *p* < 0.05, 95% CI [−0.121, −0.006]; complete mediation.
  - Techno-insecurity → quality of life: indirect effect β = −0.115, *p* < 0.01, 95% CI [−0.187, −0.043]; complete mediation.

The rest of the relationships between the technostress factors (techno-overload, techno-invasion) and the quality of life are not mediated by positive affect and negative affect; that is, hypotheses H1.1, H1.2, H1.3, H2.1, H2.2, H2.5 are not confirmed.

A significant direct effect was also identified between techno-uncertainty and quality of life (β = 0.093, *p* = 0.05, 95% CI [0, 0.186]).

As far as the total effect is concerned, in Table 2, we also find the relationships techno-insecurity–quality of life (β = −0.198, *p* < 0.05, 95% CI [−0.369,−0.027]) and techno-uncertainty–quality of life (β = 0.206, *p* = 0.01, 95% CI [0.072, 0.34]) as significant.

Analyzing the proposed model by components, we can notice the following:

- the relationship between positive affect and the quality of life is significant, positive and with a robust effect (the confidence interval does not contain the value 0), β = 0.497, *p* < 0.001, 95% CI [0.404, 0.59];
- the relationship between negative affect and the quality of life is significant, negative and with a robust effect, β= −0.414, *p* < 0.001, 95% CI [−0.515, −0.312].

The bootstrap resampling technique with 5000 replications was applied to assess the significance of the multiple mediation model, considering as significant the relationships whose 95% confidence interval does not contain the value 0 (B.-J. Kim et al., 2024; S. Liu et al., 2023; Yue et al., 2022). The results obtained after applying the bootstrap technique can be seen in Table 3. A detailed overview of the complete bootstrap results (5000 resamples) for all paths in the mediation model can be found in the Supplementary Material (Table S1).

In Table 3 we can notice that the mediation relationships described above are largely maintained. The main exception is the relationship Techno-insecurity → Positive Affect → Quality of life, which becomes insignificant.

As to the relationship Techno-uncertainty → Positive Affect → Quality of Life, it is considered significant, following the bootstrap resampling with 5000 replications, given that *p* = 0.051 and the 95% CI [0.001, 0.151] interval does not contain the value 0.

## 4. Discussion

The aim of the current study was to analyze the relationships between technostress factors (techno-overload, techno-invasion, techno-complexity, techno-insecurity, techno-uncertainty) from the Technostress creators model proposed by Ragu-Nathan et al. (2008) and the quality of life on a batch of adult Romanian citizens, a relationship mediated in turn by the mediators positive affect and negative affect.

The descriptive statistical analysis showed that the respondents registered moderate significant negative levels of techno-invasion, techno-complexity and techno-insecurity in relation to the quality of life. More specifically, the inability to delimit professional and personal time due to the ubiquitous AI technology or the perceived complexity of this technology or the perceived insecurity in relation to this technology (e.g., fear of losing one’s job, not keeping up with changes) are associated with a lower quality of life. Also, from the descriptive statistics, Table 1, we can see that technostress mainly affects (already) negative emotions (four out of five predictors of technostress are significantly positively correlated with negative affect), rather than reducing positive emotions (only one out of the five predictors of technostress–techno-complexity (r = −0.135, *p* < 0.05), is significantly negatively correlated with positive affect).

Referring to age, also from the descriptive statistics, Table 1, we can notice that older people report a better quality of life (r = 0.225, *p* < 0.001) and are less affected by technostress (techno-invasion (r = −0.19, *p* < 0.01), techno-insecurity (r = −0.188, *p* < 0.01)), but they perceive technology as more complex (r = 0.189, *p* < 0.01). Although gender and age were collected as part of the demographic data, they were not included as covariates in the mediation model, since the focus of the analysis was on psychological mechanisms—particularly the role of affect—rather than on sociodemographic influences.

The first set of hypotheses tested, H1 (H1.1–H1.5), aimed to assess the relationships that form between the technostress factors and the quality of life, in the context of mediation by the positive affect variable. Therefore, each technostress factor corresponding to each hypothesis (H1.1: Techno-overload, H1.2: Techno-invasion, H1.3: Techno-complexity, H1.4: Techno-insecurity, H1.5: Techno-uncertainty) was tested in turn, alternatively, and the following results were obtained:

- Positive affect mediates the relationship between techno-uncertainty and quality of life, contrary to our initial hypothesis H1.5, which predicted a negative association. This suggests that techno-uncertainty can foster curiosity and openness towards new technologies, enhancing positive emotions and, consequently, quality of life. This indicates that some individuals perceive technological change as an opportunity, rather than a threat, with some research even associating techno-uncertainty with increased productivity (Ismail et al., 2023). Although unexpected, this result is also confirmed by the specialized literature. Wang and Yao (2025) identified a positive association between technological uncertainty and psychological well-being. Similarly, Wang and Zhao (2023) found that technological uncertainty positively influences individuals’ attitudes toward technology and its adoption. In the same vein, Yuan et al. (2023) concluded that not all technostressors have adverse effects; for example, techno-uncertainty does not impair workplace performance.
- Positive affect mediates the relationship between techno-insecurity and the quality of life. This result supports the hypothesis H1.4, but does not confirm it clearly enough. Techno-insecurity tends to reduce positive emotions (decreases optimism, trust in technology), negatively affecting the quality of life. The result is marginally significant, indicating the tendency that techno-insecurity decreases positive affect, a situation that may have a negative impact on the quality of life. This tendency is confirmed by the specialized literature: techno-insecurity or technological insecurity has been associated with poor mental health risk and increased burnout (Yang et al., 2025), fear of losing one’s job because of technology or colleagues with better technical skills (Yeniaras & Altıniğne, 2023). The fear of losing one’s job actually represents job insecurity, a situation that affects well-being (Hellgren et al., 1999) in whose component we also find positive affect (H. Liu et al., 2023), implicitly generating a decrease in the quality of life (Piper, 2015).

It is also important to mention the fact that, after applying the bootstrap technique with 5000 replications, the relationship Techno-uncertainty → Positive Affect → Quality of life remains significant (the 95% CI [0.001, 0.151] does not contain the value 0), whereas the relationship Techno-insecurity → Positive Affect → Quality of life remains marginally significant (*p*-value = 0.069, which is relatively close to 0.05 threshold). Building on this, we can conclude that the tendency for positive affect to mediate the techno-insecurity–quality of life relationship is still present.

The second set of hypotheses tested, H2 (H2.1–H2.5), aimed to evaluate the relationships between technostress factors and the quality of life, in the context of mediation by the negative affect variable. As in the case of the first set of hypotheses, each technostress factor corresponding to each hypothesis (H2.1: Techno-overload, H2.2: Techno-invasion, H2.3: Techno-complexity, H2.4: Techno-insecurity, H2.5: Techno-uncertainty) was tested in turn, alternatively, and the following results were obtained:

- Negative affect mediates the relationship between techno-complexity and the quality of life, thus supporting hypothesis H2.3. Specifically, techno-complexity can lead to anxiety, stress and negative emotions, experiences that reduce the quality of life. In other words, the perception of technology complexity contributes to increased negative affect which, in turn, reduces the quality of life. This means that people who perceive technology as too complicated may experience frustration, anxiety, and lack of confidence in their own abilities. The specialized literature shows that the perception of low self-efficacy in using technology is associated with high stress and negative emotions (Tarafdar et al., 2019). Also, according to the literature, on the one hand, persistent negative emotions are correlated with lower life satisfaction and reduced psychological well-being (Geng et al., 2020; Tsujimoto et al., 2024), and on the other hand, the quality of life is negatively associated with stress (Clark et al., 2011; Rusli et al., 2008). The results obtained emphasize the importance of developing a positive attitude towards AI technology by resorting to psychological support with a view to managing negative affect, as well as by implementing training programs aimed at improving technical skills specific to the AI field.
- Negative affect mediates the relationship between techno-insecurity and quality of life. This result supports hypothesis H2.4. Techno-insecurity can increase fear, uncertainty and stress related to technology, experiences that reduce the quality of life. More specifically, the feeling of insecurity/uncertainty towards technology (fear of losing one’s job due to automation, for example) increases negative affect, which reduces the quality of life. According to the literature, the negative affect system generates more intense emotional reactions, per unit of stimulus, than the positive affect system—a phenomenon known as the “negativity bias” (Larsen, 2009), which means that, in this case, the quality of life is much more affected than in the situation where the relationship is mediated by positive affect, and individuals feel the negative consequences much more intensely. In other words, the fear of not being able to keep up with AI technology can lead to anxiety and psychological stress.

These relationships (Techno-complexity → Negative Affect → Quality of life; Techno-insecurity → Negative Affect → Quality of life) remain significant even after applying the bootstrap technique with 5000 replications.

The analysis of the effects on the components, in Table 1, reinforces what has already been emphasized previously and is also confirmed by the specialized literature, as follows:

- Negative affect has a significant negative effect on the quality of life (β = −0.414, *p* < 0.001). A possible interpretation of this relationship is that the higher the level of negative affect is, the lower the quality of life will be. This effect suggests that persistent negative emotions are associated with a decrease in life satisfaction (Kuppens et al., 2008; G. Wang et al., 2018).
- Positive affect has a significant positive effect on the quality of life (β = 0.497, *p* < 0.001). A possible interpretation of this relationship is that people who experience a higher level of positive affect tend to have a better quality of life. This result highlights the fact that positive affective states are predictive of well-being and life satisfaction (Cohn et al., 2009; G. Wang et al., 2018).

These results confirm that affect acts as a mediating variable between the technostress factors and the quality of life, as negative affect significantly reduces the quality of life, while positive affect significantly improves the quality of life.

In this context, we can appreciate that reducing negative affect and promoting positive affect could be effective strategies for maintaining a high quality of life, even in contexts of technostress generated by AI.

- Techno-insecurity significantly increases negative affect (β = 0.278, *p* < 0.001), highlighting that fear of technological changes produced by AI is a major stressor, while techno-uncertainty increases positive affect (β = 0.148, *p* < 0.05), confirming the idea that uncertainty can be perceived as a learning opportunity. This aspect is also reinforced by the only marginally significant direct relationship in the model (between technostress factors and the quality of life), but very weak: techno-uncertainty can positively predict the quality of life (β = 0.093, *p* = 0.05).
- Techno-complexity also increases negative affect (β = 0.153, *p* < 0.05), highlighting, on the one hand, that perceiving AI technology as difficult can induce anxiety and stress (Ragu-Nathan et al., 2008; Tarafdar et al., 2019), which explains why negative affect increases, and on the other hand, AI complexity can be perceived by people as a threat to their skills (Lazarus & Folkman, 1984), leading to stress and negative emotions.
- Techno-insecurity decreases positive affect (β = −0.176, *p* < 0.05). This situation occurs when people feel uncertainty about their professional future, decreasing motivation and sense of control (Trevor-Roberts, 2006), implicitly reducing positive emotions.

The only significant direct effect identified in the model—both prior to and after applying the bootstrap technique—was the relationship between techno-uncertainty and quality of life (β = 0.093, *p* = 0.045). This suggests that techno-uncertainty may not only exert an indirect influence through affective mechanisms, but can also have a standalone, positive impact. One possible explanation is that uncertainty related to AI may trigger cognitive engagement and adaptive coping strategies, such as proactive learning or future-oriented thinking, which contribute directly to a better perception of life quality (Gajos & Mamykina, 2022; Tong et al., 2025). From this perspective, the effect may reflect a broader psychological process beyond emotion—namely, the individual’s capacity for resilience and cognitive reframing in the face of evolving technologies.

The overall effects (including both direct and indirect effects) of the analyzed model are represented by the relationships:

- Techno-insecurity has a significant overall negative effect on the quality of life (β = −0.198, *p* < 0.05), highlighting the fact that technological insecurity induced by the development and implementation of AI is the strongest risk factor for the quality of life.
- Techno-uncertainty has a significant positive overall effect (β = 0.206, *p* < 0.05), indicating that the technological uncertainty that arises with AI can also have beneficial aspects.

Beyond theoretical relevance, these findings also offer practical insights for organizational environments. Given the mediating role of affect, organizations should consider not only the technical aspects of AI integration, but also its emotional impact on employees. Interventions such as resilience-building programs, emotional regulation training, and psychological support services may help employees better manage stress and maintain emotional balance. Furthermore, ensuring transparent communication and adequate training can mitigate the risks of perceived insecurity or complexity related to AI. By fostering a climate of clarity, support, and emotional well-being, organizations can enhance employee adaptation and protect overall quality of life during technological transitions.

When the machine imitates human thinking and actions through data analysis, it can be called AI (Xia, 2023). Although the technostress factors have been studied in the specialized literature in relation to the current technologies, such as smartphone or digital platforms (Bondanini et al., 2020; La Torre et al., 2020), studies focusing specifically on AI technology remain limited. Both in terms of operation and increased performance, AI technology is unprecedented in the history of technology, generating, in addition to benefits, fear and uncertainties. The future is taking shape right now; however, what will truly define the quality of life is not the technology itself, but the emotions we experience, the balance between positive and negative affect.

## 5. Limitations, Future Directions and Final Remarks

Even though the current study is among the first studies of this kind conducted on the adult population of Romania and brings valuable information, in a world ”conquered” by uncertainties and fears, it is not without limitations.

The current descriptive, exploratory, differential, and correlational study relied on self-administered questionnaires, which, although appropriate for the design and context, may still be susceptible to other types of response bias (El-Zoghby et al., 2024). For instance, participants may have responded in socially desirable ways or may have experienced recall bias when reporting past emotions or behaviors. Future studies could consider using complementary methods, such as physiological measures or behavioral data, to enhance objectivity and reduce such potential biases.

Secondly, the present study employed a cross-sectional design, which limits the ability to draw conclusions about causal direction among the variables examined (Khan et al., 2023; Y. Wang et al., 2022). This is particularly relevant for mediation analysis, where the assumption of temporal ordering between predictor, mediator, and outcome cannot be verified. As extensively discussed in the methodological literature (Maxwell & Cole, 2007; Mitchell & Maxwell, 2013; O’Laughlin et al., 2018), testing mediation on cross-sectional data may lead to biased or inconclusive interpretations. Therefore, the current findings should be viewed as preliminary and correlational. Future studies are encouraged to adopt longitudinal designs that allow for more robust causal inferences.

Although the mediation model in the current study—incorporating the five technostress factors, positive affect, and negative affect—explains 51.8% of the variance in quality of life, future research could consider integrating additional variables to account for the remaining 48.2%, thereby further enhancing the explanatory power of the model.

Another limitation of the current research is that there was no differentiation between the types of AI information systems and no differentiation regarding the use of AI, for example, between the professional and personal spheres. Therefore, future studies can perform similar analyses, but taking into account the elements mentioned above and not included in this model.

Furthermore, the study did not specifically assess participants’ background knowledge or training in AI or ICT. This is an important factor to consider, as prior knowledge may moderate the psychological impact of AI. To address this, future research is encouraged to include such measures for a more nuanced understanding of technostress responses.

An additional limitation of the present study concerns the generalizability of the findings. Although the sample included a diverse range of participants in terms of age, gender, education, professional status, and area of residence (urban vs. rural), it was not designed to be nationally representative of the Romanian adult population. The sampling method relied on voluntary participation and online distribution, which may have introduced self-selection bias. Therefore, while the results offer valuable insights into the psychological effects of AI-related technostress, they should be interpreted with caution when generalizing to broader populations. Future studies are encouraged to replicate these findings using larger, probability-based samples.

Finally, we reiterate that AI is a key factor in transforming industries, improving innovation, efficiency and company development (Gao et al., 2025). However, during this transformation, it may be difficult to understand how AI will change the way work will be performed (Aromaa et al., 2025) in the not too remote future. Therefore, the context is favorable to fears and uncertainties, especially in relation to the professional field, namely the amplification of negative emotions and the diminution of positive ones, influencing people’s quality of life. Nevertheless, it is necessary to emphasize, as previously mentioned, that it is not the development and implementation of AI technology that directly influences the quality of life. The real influence comes from the emotions felt in this context, namely what humans can actually control and improve, both through specific training for AI technology and by accessing psychological services.

## Figures and Tables

**Table 1:** Descriptive statistics and correlations between the investigated variables.

	Mean	SD	1	2	3	4	5	6	7	8	9	10
1. Techno-overload	12.92	5.50	—									
2. Techno-invasion	5.50	3.02	0.673 ***	—								
3. Techno-complexity	11.47	4.77	0.391 ***	0.383 ***	—							
4. Techno-insecurity	9.29	4.24	0.577 ***	0.589 ***	0.425 ***	—						
5. Techno-uncertainty	11.33	4.80	0.288 ***	0.208 **	0.091	0.304 ***	—					
6. Quality of Life	86.37	16.47	−0.117	−0.186 **	−0.205 **	−0.216 **	0.129	—				
7. Negative Affect	20.89	8.06	0.288 ***	0.347 ***	0.317 ***	0.398 ***	0.033	−0.584 ***	—			
8. Positive Affect	33.30	7.24	0.035	−0.026	−0.135 *	−0.103	0.124	0.638 ***	−0.317 ***	—		
9. Gender			−0.023	0.02	0.225 ***	0.024	0.001	0.076	0.158 *	0.074	—	
10. Age	36.15	11.92	−0.133	−0.19 **	0.189 **	−0.188 **	−0.09	0.225 ***	−0.174 *	0.144 *	0.37 ***	—

Note: * *p* < 0.05, ** *p* < 0.01, *** *p* < 0.001, SD = Standard Deviation, 1 = Techno-overload, 2 = Techno-invasion, 3 = Techno-complexity, 4 = Techno-insecurity, 5 = Techno-uncertainty, 6 = Quality of Life, 7 = Negative Affect, 8 = Positive Affect, 9 = Gender, 10 = Age.

**Table 2:** Results of the mediation analysis.

				95% CI (a)			
Type	Effect	Estimate (β)	SE	Lower	Upper	z	*p*
Indirect	Techno-Overload ⇒ Negative Affect ⇒ Quality of Life	0.002	0.036	−0.069	0.073	0.059	0.953
	Techno-Overload ⇒ Positive Affect ⇒ Quality of Life	0.072	0.048	−0.022	0.166	1.501	0.133
	Techno-Invasion ⇒ Negative Affect ⇒ Quality of Life	−0.061	0.037	−0.134	0.011	−1.66	0.097
	Techno-Invasion ⇒ Positive Affect ⇒ Quality of Life	0	0.047	−0.093	0.092	−0.003	0.998
	**Techno-Complexity ⇒ Negative Affect ⇒ Quality of Life**	**−0.063**	**0.029**	**−0.121**	**−0.006**	**−2.149**	**0.032**
	Techno-Complexity ⇒ Positive Affect ⇒ Quality of Life	−0.065	0.038	−0.138	0.009	−1.715	0.086
	**Techno-Insecurity ⇒ Negative Affect ⇒ Quality of Life**	**−0.115**	**0.037**	**−0.187**	**−0.043**	**−3.116**	**0.002**
	**Techno-Insecurity ⇒ Positive Affect ⇒ Quality of Life**	**−0.087**	**0.045**	**−0.176**	**0.001**	**−1.935**	**0.053**
	Techno-Uncertainty ⇒ Negative Affect ⇒ Quality of Life	0.039	0.027	−0.014	0.092	1.443	0.149
	**Techno-Uncertainty ⇒ Positive Affect ⇒ Quality of Life**	**0.073**	**0.036**	**0.004**	**0.143**	**2.067**	**0.039**
Component	Techno-Overload ⇒ Negative Affect	−0.005	0.088	−0.177	0.166	−0.059	0.953
	**Negative Affect ⇒ Quality of Life**	**−0.414**	**0.052**	**−0.515**	**−0.312**	**−7.998**	**<** **0.001**
	Techno-Overload ⇒ Positive Affect	0.145	0.095	−0.042	0.331	1.517	0.129
	**Positive Affect ⇒ Quality of Life**	**0.497**	**0.048**	**0.404**	**0.59**	**10.457**	**<** **0.001**
	Techno-Invasion ⇒ Negative Affect	0.148	0.087	−0.023	0.319	1.697	0.09
	Techno-Invasion ⇒ Positive Affect	0	0.095	−0.186	0.186	−0.003	0.998
	**Techno-Complexity ⇒ Negative Affect**	**0.153**	**0.069**	**0.019**	**0.288**	**2.231**	**0.026**
	Techno-Complexity ⇒ Positive Affect	−0.13	0.075	−0.276	0.017	−1.738	0.082
	**Techno-Insecurity ⇒ Negative Affect**	**0.278**	**0.082**	**0.117**	**0.439**	**3.384**	**<** **0.001**
	**Techno-Insecurity ⇒ Positive Affect**	**−0.176**	**0.089**	**−0.351**	**−0.001**	**−1.969**	**0.049**
	Techno-Uncertainty ⇒ Negative Affect	−0.094	0.064	−0.221	0.032	−1.467	0.142
	**Techno-Uncertainty ⇒ Positive Affect**	**0.148**	**0.07**	**0.01**	**0.285**	**2.109**	**0.035**
Direct	Techno-Overload ⇒ Quality of Life	−0.019	0.064	−0.145	0.106	−0.303	0.762
	Techno-Invasion ⇒ Quality of Life	−0.04	0.064	−0.166	0.085	−0.632	0.527
	Techno-Complexity ⇒ Quality of Life	0.006	0.051	−0.094	0.105	0.115	0.908
	Techno-Insecurity ⇒ Quality of Life	0.004	0.062	−0.116	0.125	0.072	0.943
	**Techno-Uncertainty ⇒ Quality of Life**	**0.093**	**0.047**	**0**	**0.186**	**1.963**	**0.05**
Total	Techno-Overload ⇒ Quality of Life	0.055	0.093	−0.128	0.237	0.586	0.558
	Techno-Invasion ⇒ Quality of Life	−0.102	0.093	−0.284	0.08	−1.098	0.272
	Techno-Complexity ⇒ Quality of Life	−0.122	0.073	−0.265	0.021	−1.672	0.095
	**Techno-Insecurity ⇒ Quality of Life**	**−0.198**	**0.087**	**−0.369**	**−0.027**	**−2.268**	**0.023**
	**Techno-Uncertainty ⇒ Quality of Life**	**0.206**	**0.068**	**0.072**	**0.34**	**3.005**	**0.003**

Note. All paths represent standardized estimates obtained after z-scoring the variables. Values in bold denote statistically significant findings (*p* < 0.05, *p* < 0.01, or *p* < 0.001). Abbreviations: CI = Confidence Interval; β = Standardized coefficient (Beta); SE = Standard Error; z = z-statistic; *p* = *p*-value (statistical significance).

**Table 3:** The results following the application of the bootstrap technique.

				95% CI (a)			
Type	Effect	Estimate (β)	SE	Lower	Upper	z	*p*
Indirect	**Techno-Complexity ⇒ Negative Affect ⇒ Quality of Life**	**−0.063**	**0.031**	**−0.126**	**−0.005**	**−2.051**	**0.04**
	**Techno-Insecurity ⇒ Negative Affect ⇒ Quality of Life**	**−0.115**	**0.039**	**−0.196**	**−0.041**	**−2.911**	**0.004**
	Techno-Insecurity ⇒ Positive Affect ⇒ Quality of Life	−0.087	0.048	−0.183	0.007	−1.82	0.069
	**Techno-Uncertainty ⇒ Positive Affect ⇒ Quality of Life**	**0.073**	**0.038**	**0.001**	**0.151**	**1.953**	**0.051**
Component	**Negative Affect ⇒ Quality of Life**	**−0.414**	**0.052**	**−0.517**	**−0.313**	**−7.934**	**<0.001**
	**Positive Affect ⇒ Quality of Life**	**0.497**	**0.055**	**0.386**	**0.602**	**9.07**	**<0.001**
	**Techno-Complexity ⇒ Negative Affect**	**0.153**	**0.071**	**0.012**	**0.292**	**2.152**	**0.031**
	**Techno-Insecurity ⇒ Negative Affect**	**0.278**	**0.089**	**0.104**	**0.452**	**3.119**	**0.002**
	Techno-Insecurity ⇒ Positive Affect	−0.176	0.099	−0.378	0.013	−1.774	0.076
	**Techno-Uncertainty ⇒ Positive Affect**	**0.148**	**0.074**	**0.003**	**0.294**	**1.984**	**0.047**
Direct	**Techno-Uncertainty ⇒ Quality of Life**	**0.093**	**0.047**	**0.009**	**0.189**	**2.002**	**0.045**
Total	Techno-Insecurity ⇒ Quality of Life	−0.198	0.103	−0.395	0.004	−1.917	0.055
	**Techno-Uncertainty ⇒ Quality of Life**	**0.206**	**0.074**	**0.064**	**0.348**	**2.795**	**0.005**

Note. All estimates represent standardized coefficients (β) obtained after z-scoring the variables. CI = Confidence Interval; SE = Standard Error; z = z-statistic; *p* = *p*-value (statistical significance). This table displays only the significant and marginally significant results following the 5000-resample bootstrap procedure. Values in bold denote statistically significant findings (*p* < 0.05, *p* < 0.01, or *p* < 0.001).

## Data Availability

The original contributions presented in this study are included in the article; further inquiries can be directed to the corresponding author.

## Supplementary Materials

The following supporting information can be downloaded at: https://www.mdpi.com/article/10.3390/bs15040552/s1. Table S1. Complete bootstrap results for all effects in the mediation model (5000 resamples).

## Abbreviations

The following abbreviations are used in this manuscript:

AI	Artificial Intelligence
β	Beta (the standardized path coefficient between latent variables)
CI	Confidence Interval
F	Function
GLM	General Linear Model
H	Hypothesis
ICT	Information and Communication Technology
M	Mean
*p*	*p*-value (statistical significance)
PANAS-X	Positive and Negative Affect Scales, extended form
QOLS	Quality of Life Scale
r	Pearson correlation coefficient
SD	Standard Deviation
SE	Standard Error
VIF	Variance Inflation Factor
z	z-statistic
