# Supplementary material for: The Impact of Technostress Generated by Artificial Intelligence on the Quality of Life: The Mediating Role of Positive and Negative Affect
Source: Behav Sci (Basel). 2025 Apr 19;15(4):552. doi: 10.3390/bs15040552 (PMC12024279; doi:10.3390/bs15040552)
Supplement: Supplementary file 1 [file behavsci-15-00552-s001.zip › behavsci-3577885-supplementary.pdf]

SUPPLEMENTAL MATERIAL

Table S1. Complete bootstrap results for all effects in the mediation model (5000 resamples)

Type	Effect	Estimate (β)	SE	95% CI (a) Lower	Upper	z	*p*
Indirect	Techno-Overload ⇒ Negative Affect ⇒ Quality of Life	0.002	0.038	-0.071	0.079	0.056	0.955
	Techno-Overload ⇒ Positive Affect ⇒ Quality of Life	0.072	0.058	-0.035	0.193	1.24	0.215
	Techno-Invasion ⇒ Negative Affect ⇒ Quality of Life	-0.061	0.037	-0.14	0.005	-1.669	0.095
	Techno-Invasion ⇒ Positive Affect ⇒ Quality of Life	0	0.054	-0.115	0.097	-0.002	0.998
	**Techno-Complexity ⇒ Negative Affect ⇒ Quality of Life**	**-0.063**	**0.031**	**-0.126**	**-0.005**	**-2.051**	**0.04**
	Techno-Complexity ⇒ Positive Affect ⇒ Quality of Life	-0.065	0.038	-0.143	0.008	-1.691	0.091
	**Techno-Insecurity ⇒ Negative Affect ⇒ Quality of Life**	**-0.115**	**0.039**	**-0.196**	**-0.041**	**-2.911**	**0.004**
	Techno-Insecurity ⇒ Positive Affect ⇒ Quality of Life	-0.087	0.048	-0.183	0.007	-1.82	0.069
	Techno-Uncertainty ⇒ Negative Affect ⇒ Quality of Life	0.039	0.027	-0.013	0.094	1.438	0.151
	**Techno-Uncertainty ⇒ Positive Affect ⇒ Quality of Life**	**0.073**	**0.038**	**0.001**	**0.151**	**1.953**	**0.051**
Component	Techno-Overload ⇒ Negative Affect	-0.005	0.091	-0.185	0.171	-0.057	0.955
	**Negative Affect ⇒ Quality of Life**	**-0.414**	**0.052**	**-0.517**	**-0.313**	**-7.934**	**< .001**
	Techno-Overload ⇒ Positive Affect	0.145	0.114	-0.071	0.375	1.263	0.206
	**Positive Affect ⇒ Quality of Life**	**0.497**	**0.055**	**0.386**	**0.602**	**9.07**	**< .001**
	Techno-Invasion ⇒ Negative Affect	0.148	0.084	-0.013	0.317	1.752	0.08
	Techno-Invasion ⇒ Positive Affect	0	0.109	-0.226	0.198	-0.002	0.998
	**Techno-Complexity ⇒ Negative Affect**	**0.153**	**0.071**	**0.012**	**0.292**	**2.152**	**0.031**
	Techno-Complexity ⇒ Positive Affect	-0.13	0.076	-0.277	0.016	-1.72	0.085
	**Techno-Insecurity ⇒ Negative Affect**	**0.278**	**0.089**	**0.104**	**0.452**	**3.119**	**0.002**
	Techno-Insecurity ⇒ Positive Affect	-0.176	0.099	-0.378	0.013	-1.774	0.076
	Techno-Uncertainty ⇒ Negative Affect	-0.094	0.063	-0.217	0.031	-1.488	0.137
	**Techno-Uncertainty ⇒ Positive Affect**	**0.148**	**0.074**	**0.003**	**0.294**	**1.984**	**0.047**
Direct	Techno-Overload ⇒ Quality of Life	-0.019	0.07	-0.152	0.121	-0.275	0.783
	Techno-Invasion ⇒ Quality of Life	-0.04	0.073	-0.193	0.098	-0.553	0.58
	Techno-Complexity ⇒ Quality of Life	0.006	0.051	-0.094	0.108	0.115	0.909
	Techno-Insecurity ⇒ Quality of Life	0.004	0.068	-0.132	0.133	0.065	0.948
	**Techno-Uncertainty ⇒ Quality of Life**	**0.093**	**0.047**	**0.009**	**0.189**	**2.002**	**0.045**
Total	Techno-Overload ⇒ Quality of Life	0.055	0.108	-0.159	0.269	0.503	0.615
	Techno-Invasion ⇒ Quality of Life	-0.102	0.104	-0.322	0.082	-0.982	0.326
	Techno-Complexity ⇒ Quality of Life	-0.122	0.073	-0.263	0.024	-1.669	0.095
	**Techno-Insecurity ⇒ Quality of Life**	**-0.198**	**0.103**	**-0.395**	**0.004**	**-1.917**	**0.055**
	**Techno-Uncertainty ⇒ Quality of Life**	**0.206**	**0.074**	**0.064**	**0.348**	**2.795**	**0.005**

Note. All estimates represent standardized coefficients (β) obtained after z-scoring the variables. CI = Confidence Interval; SE = Standard Error; z = z-statistic; p = p-value (statistical significance). Values in bold indicate statistically significant results ($p < 0.05$, $p < 0.01$, or $p < 0.001$), or marginally significant findings.
